# Isolated Langerhans Cell Histiocytosis of the Thyroid in an Adult Female: One-Year Followup

**DOI:** 10.1155/2011/898302

**Published:** 2011-03-06

**Authors:** Ramon Vilallonga, Andrea Ciudin, José Manuel Fort, Juan Antonio Baena, Oscar Gonzalez, Manuel Armengol, Jordi Mesa, Mari Carmen Ruiz Marcellán

**Affiliations:** ^1^Endocrine, Bariatric and Metabolic Unit, General Surgery Department, University Hospital Vall d'Hebron, 08035 Barcelona, Spain; ^2^Endocrinology Department, University Hospital Vall d'Hebron, 08035 Barcelona, Spain; ^3^General Surgery Department, University Hospital Vall d'Hebron, Passeig de la Vall d'Hebron 119-129, 08035 Barcelona, Spain; ^4^Pathology Department, University Hospital Vall d'Hebron, 08035 Barcelona, Spain

## Abstract

Thyroid gland involvement as the unique presentation of Langerhans cell histiocytosis is a rare phenomenon that can result in misdiagnosis. We report a case of Langerhans cell histiocytosis (LCH) presenting as a thyroid mass. It is a 52-year-old woman who presented an enlarged, diffusely firm, nontender, nonmobile, and not particularly nodular thyroid gland with mild compressive symptoms. Ultrasound and fine-needle aspiration showed a unique right node with benign signs. Patient was referred to our Ambulatory Surgery Department, where a hemithyroidectomy was performed. Histologic evaluation of the right thyroid gland revealed an involvement by LCH, confirmed by immunohistochemical analysis showing Langerhans cells that were positive for CD1a. LCH was a completely incidental occult finding apparent only after surgical resection and examination of the gland. Patient was evaluated, and no evidence of systemic affectation was found. LCH can rarely involve the thyroid gland in adults. Few cases have been reported in the literature. Most patients had evidence of LCH involving other anatomic sites.

## 1. Introduction

Langerhans cell histiocytosis (LCH) involving the thyroid is a very rare condition, which presents typically with involvement of bone, lung, skin, the hypothalamus/posterior pituitary gland, lymph nodes, and multiple sites. [1] Few cases have been reported of thyroid gland infiltration by LCH as isolated involvement. [2] We do present a case of isolated thyroid involvement by LCH.

## 2. Case Report

A 52-year-old woman who presented with an enlarging thyroid mass was referred to our Surgical Department for evaluation. Physical examination of the patient revealed an enlarged, diffusely firm, nontender, nonmobile, and not particularly nodular thyroid gland with mild compressive symptoms. This thyroid gland revealed no tenderness, and the overlying skin was not erythematous. Preoperative endocrine evaluation revealed normal levels for thyroxine (T4; 7.2 *μ*g/dL, normal range 4.9–13), tri-iodothyronine (T3; 109 ng/dL, normal range 80–185), and thyroid-stimulating hormone (TSH; 3. 99 *μ*U/mL, normal range 0.6–5.5). 

Ultrasound showed diffusely, hypoechoic thyroid with dimensions of 36 × 20 × 16 mm on the right and 36 × 16 × 17 mm on the left. A unique nodule with dimensions was found in the right lobe. The ultrasound could not see any other lesions. A fine-needle aspiration confirmed the diagnosis of hyperplasic nodule. The patient then underwent a right hemithyroidectomy to remove this enlarging and compressing node in the Ambulatory Surgery Department.

## 3. Pathological Findings

The thyroid gland specimens are routinely processed, formalin-fixed, and paraffin-embedded; histologic sections are stained with hematoxylin and eosin (H&E). Immunohistochemical stains are performed on representative formalin-fixed paraffin sections at our institution using a biotin-streptavidin method with appropriate controls. The specimen was assessed for expression of CD1a, S-100 protein, leukocyte common antigen (LCA, CD45). Other studies included thyroglobulin.

The right thyroid gland removed was 14.5 g and measured 30 × 25 × 25 mm. The node was occupying more than 3 of the 4 parts of the right lobe. The node measured 30 × 20 × 20 mm and appeared to be an adenoma. All thyroid enlargement was due to the hyperplasic node. At the isthmus zone, a central white area was observed to be measured 10 × 6 × 5 mm.

**Figure 1:**
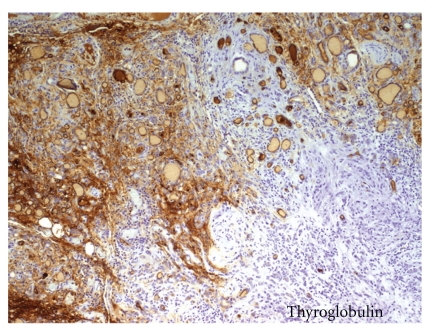
Focus of polymorph infiltrate and histiocytic negative for thyroglobulin. Normal positives thyroid cells peripherally. (Immunohistochemical Biotin-streptavidin Technique. 100x)

**Figure 2:**
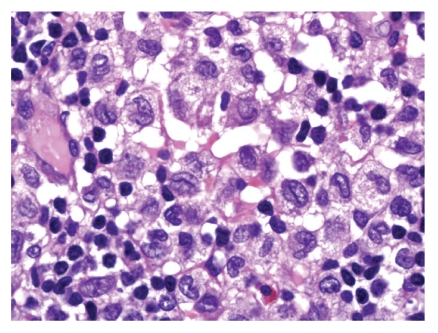
HE of the focus of histiocytes. Focus histiocytic infiltrate with coffee bean morphology, lymphoid infiltrate with eosinophils. (H.E. 200x)

**Figure 3:**
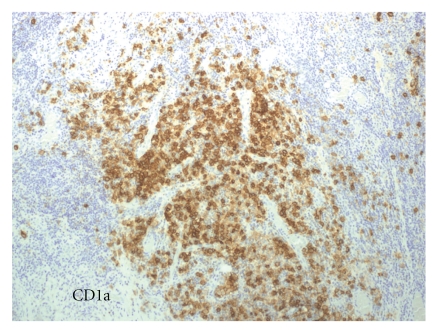
CD1a-positive histiocytes. (Immunohistochemical streptavidin-biotin Technique. 100x)

Histological evaluation revealed LCH. On low magnification, there was no evidence of a distinct, well-circumscribed mass. Sheets of discohesive mononuclear cells separated large areas of normal appearing thyroid follicles. On closer examination, these cells contained a moderate to abundant amount of pale to eosinophilic cytoplasm with deeply clefted, slightly eccentric nuclei characteristic of LCs. In focal areas, the LC infiltrate appeared to merge with the adjacent follicular epithelium, and individual tumour cells were seen within thyroid epithelium and in follicle lumina (Figure 1). The LCs were negative for epithelial membrane antigen, thyroglobulin (Figure 2). The LCs were strongly positive for CD1a with a diffuse cytoplasmic pattern of staining (Figure 3).

This LCH appeared to be an incidental almost occult finding in a thyroid that was resected for an enlarging, compressing thyroid adenoma. A retrospectively review of the preoperative ultrasound images with the radiologist could not detect the incidental finding of the LCH node.

Following surgery, a whole-body bone scan and skull X-ray were done, followed by bone marrow aspiration and examination, all of which revealed no definitive evidence of any disease involvement. Urine analysis, a complete blood cells count, erythrocyte sedimentation rate, and serum biochemistry (electrolytes, alkaline phosphatase, and liver function) all yielded results within the normal ranges. At this stage, a definitive diagnosis of isolated LCH of the thyroid was made. After 1-year followup, no evidence of systemic disease has been proven.

## 4. Discussion

LCH is a rare disease with an incidence rate of 4.0–5.4 per 1 million individuals, and the most common endocrinological manifestation of classical LCH is associated with posterior pituitary involvement presenting as diabetes insipidus [3]. Some other manifestations such as hypothalamic/pituitary axis disturbance and anterior pituitary deficiency can be less frequently observed, resulting in secondary or tertiary hypothyroidism [4]. Also the condition typically presents with the involvement of bone, lung, skin, lymph nodes, and multiple sites [5]. However, LCH involving the thyroid gland is extremely rare, and there are very few reported cases, especially when it is a unique location [6]. Most of the cases present as a diffuse or nodular thyroid enlargement and as multisystemic disease, and only few cases were reported as isolated involvement of the thyroid by LCH [7, 8]. Because LCH involving the thyroid gland is rare, it is not really possible to comment on the frequency of such completely occult lesions from the available literature.

Thyroid involvement is more common in adults and has a relatively indolent course. Thyroid involvement of LCH can be indistinguishable from other thyroid disorders presenting with goiter. It is important to distinguish isolated thyroid LCH from multisystemic cases because single organ involvement is associated with an excellent survival of close to 100% [9]. For this reason a complete study in order to diagnose a systemic affection is important. 

However, cases have been reported, where LCH has been initially misdiagnosed as poorly differentiated carcinoma of the thyroid [10]. 

Therefore, diagnosing these patients is quite challenging for a clinician [7–9]. Not only the physical signs but also the thyroid hormone status, antithyroid antibodies, sonographic and scintigraphic findings may be similar [11–13]. Physical examination usually reveals a multinodular or a diffusely enlarged thyroid as in our patients. They tend to present with a palpable nodule or in other cases as a diffusely enlarged goiter of one or two lobes [9]; this nonspecific presentation is easily confused with far more common benign goiters or thyroid neoplasms [7, 9]. 

Antithyroid antibodies, especially antithyroglobulin antibody, may be elevated. However, elevation of antimicrosomal antibodies, as detected in our second case, has not been documented before. This finding reminds us to keep primary thyroid LCH in mind, as well as thyroid carcinoma and autoimmune thyroiditis, in the differential diagnosis of thyroid disorders presenting with multinodular goiter [14].

 Some cases have been reported, where LCH has been initially misdiagnosed as poorly differentiated carcinoma of the thyroid. According to the literature, primary LCH and carcinoma of the thyroid were synchronously reported in adults [12, 13]. On review of previous biopsies and when clinical suspicion of LCH is high, histologic support for the diagnosis of LCH can usually be found. 

For the diagnosis, ultrasonography and fine-needle aspiration are first-line modalities for the workup of thyromegaly [15], when large histiocytes with abundant cytoplasm interspersed in a background of lymphocytes and eosinophils are seen after FNA examination in the typical presentation of LCH [6]. According to this technique, thyroid fine-needle aspiration is useful in establishing the diagnosis, but Langerhans cells are occasionally misdiagnosed as atypical follicular epithelial cells and others. Also, as mentioned above, papillary carcinoma has been reported to coexist with LCH within the thyroid gland [9]. 

Surgical treatment is the suitable option. For localized LCH to the thyroid, resection of LCH by subtotal, near total, or total thyroidectomy is the treatment of choice [7, 9]. No date has been able to show that it is necessary to perform the systemic chemotherapy; however, additional investigations such as thoracic CT, whole-body bone scintigraphy, and abdominal ultrasonography, as well as prolonged followup, are suggested [9]. 

## 5. Conclusion

In conclusion, we should consider that isolated LCH of the thyroid is quite rare during infancy and should be considered in the differential diagnosis. In adults, systemic LCH affection has to been studied. A local excision of the thyroid is the treatment of choice, and prolonged followup is recommended in these patients.
